# Associations of Chronic Hepatitis B and Nonalcoholic Fatty Liver Diseases with New-Onset Metabolic Syndrome in Military Personnel before Midlife: A Cohort Study

**DOI:** 10.2174/0118715303323078241022050619

**Published:** 2025-01-09

**Authors:** Kun-Zhe Tsai, Pang-Yen Liu, Yen-Chen Lin, Chen-Ming Huang, Hui-Shang Wang, Gen-Min Lin

**Affiliations:** 1 Department of Medicine, Hualien Armed Forces General Hospital, Hualien City, Taiwan;; 2 Department of Stomatology of Periodontology, Mackay Memorial Hospital, Taipei, Taiwan;; 3 Department of Periodontology, School of Dentistry, National Defense Medical Center and Tri-Service General Hospital, Taipei, Taiwan;; 4 Department of Medicine, Tri-Service General Hospital, National Defense Medical Center, Taipei, Taiwan;; 5 Department of Medicine, Linkou Chang Gung Memorial Hospital, Taoyuan, Taiwan;; 6 Department of Cardiovascular Surgery, Mennonite Christian Hospital, Hualien City, Taiwan;; 7 Division of Cardiology, Department of Internal Medicine, Hualien Tzu Chi General Hospital, Hualien City, Taiwan

**Keywords:** Chronic hepatitis B, metabolic syndrome, nonalcoholic fatty liver disease, nonalcoholic steatohepatitis, young adults, military health

## Abstract

**Background:**

Hepatic inflammation, *e.g.*, Nonalcoholic Fatty Liver Diseases (NAFLD) and the severe form of steatohepatitis (NASH), has been associated with a higher risk of MetS in the general population.

**Aims:**

This study aimed to investigate the associations of chronic hepatitis B (CHB) and fatty liver diseases with the incidence of metabolic syndrome (MetS) in young adults, which have not been verified before.

**Objective:**

The associations between NAFLD, NASH, and CHB and the incidence of new-onset MetS remain inconclusive in young adults.

**Methods:**

This cohort study included 2,614 military personnel aged 18-39 years who were free of baseline MetS in 2014 and were followed for the incidence of MetS in each annual health examination until the end of 2020. CHB was defined by the presence of the hepatitis B surface antigen with an established diagnosis history. NAFLD and NASH were defined by the ultrasound finding with an elevated alanine transaminase (27-53.9 and ≥54 U/L in men and 15-29.9 and ≥30 U/L in women, respectively). MetS was defined based on the International Diabetes Federation criteria. Multivariable Cox regression analysis was used to determine the associations between hepatitis and incident MetS.

**Results:**

During a mean follow-up of 6.0 years, 582 new-onset MetS cases occurred (22.3%). NAFLD and NASH were associated with a greater risk of new-onset MetS (hazard ratios (HRs) and 95% confidence intervals: 1.47 (1.21-1.79) and 1.66 (1.16-2.39), respectively), while no association for CHB was found (HR: 1.31 (0.88-1.96)).

**Conclusion:**

This study found that NAFLD and NASH, while not CHB, were independent risk factors of new-onset MetS with adjustments for potential covariates, *e.g.*, physical activity and fitness in young adults.

## 1 INTRODUCTION

Metabolic syndrome (MetS), a complex of cardiovascular disease risk factors rather than a single ailment, is characterized by central obesity, hypertension, dyslipidemia, and insulin resistance [1, 2]. In the Asia-Pacific region, MetS affects nearly one-fifth of the adult population, with an upward trend in prevalence [3]. With successful global control of numerous traditional infectious diseases, non-communicable diseases, such as MetS, have emerged as the main contributors to global morbidity and mortality [4].

The pathogenesis of MetS seems to be related to the presence of multiple factors, including inflammation [5]. The liver plays a pivotal role in the metabolism of glucose and lipids. A large body of studies have clarified a connection between chronic hepatitis and metabolic derangement [5-8]. In Asia, both hepatic steatosis and chronic viral hepatitis B (CHB) emerge as prevalent chronic liver diseases [8]. Nonalcoholic fatty liver disease (NAFLD) and the severe form of steatohepatitis (NASH) have been noted as the potential risk markers of MetS [6]. However, the association between CHB infection and MetS remains inconclusive [6]. Although active viral hepatitis, *e.g.,* hepatitis C, can cause hepatic inflammation, possibly promoting the development of MetS, evidence for the association of CHB with incident MetS is inconsistent as some studies reported a sex difference in the association [9], and a few meta-analyses concluded CHB as a protective factor of MetS, especially in young adults [6, 10]. Most of the studies were cross-sectional studies and were limited by a lack of information on baseline physical activity (PA) and fitness [11-13], which are crucial confounders for the development of MetS. Therefore, this study aimed to examine the associations of NASH, NAFLD, and CHB with the incidence of new-onset MetS among military personnel by including detailed information on physical activity and fitness status.

## 2 METHODS

### 2.1 Study Population

This cohort study included 4,080 military men and women aged 18 to 50 years, without baseline diabetes mellitus and taking any antihypertensive and lipid-lowering medications from the Cardiorespiratory Fitness and Health in Eastern Armed Forces (CHIEF) in Hualien County, Taiwan, in 2014 [14]. The CHIEF study was a prospective longitudinal study designed to find the correlates and associations of physical activity and fitness with the prevalence and incidence of cardiometabolic comorbidities among military young adults. This study has been described in detail in the previous studies [15-18].

The exclusion criteria included those who had baseline MetS, age ≥40 years, a significant increase in serum alanine transaminase (ALT) (≥ 4 x the suggested upper reference (27 U/L) in men =108 U/L) [19], and those lost to follow-up. This study adhered to the principles of the Declaration of *Helsinki*, and the study protocol and design were reviewed and approved by the Ethical Review Board of the Mennonite Christian Hospital (No. 16-05-008) in Hualien City, Taiwan. All participants provided written informed consent.

### 2.2 Annual Health Examinations (2014-2020)

Waist circumference, body weight, and body height were measured during the visit. Body mass index (BMI) was calculated using the following equation: BMI = body weight (kg)/(height (m))^2^. Overweight and obesity were categorized as BMI of 25.0 kg/m^2^ - 29.9 kg/m^2^ and ≥30 kg/m^2^, respectively, according to the criteria established by the World Health Organization [20].

The resting blood pressure (BP) of each participant was recorded once while seated, using an automatic BP device applied to the right arm (FT201 Parama-Tech Co., Ltd, Fukuoka, Japan) by the oscillometric method. In cases where the initial BP level was ≥ 130/80 mmHg, a second measurement was taken after a 15-minute rest period. The final BP level was determined as the average of the initial and second BP readings [21].

Serum concentrations of total cholesterol, low-density lipoprotein cholesterol (LDL-C), high-density lipoprotein cholesterol (HDL-C), triglycerides, uric acid, fasting glucose, ALT, and aspartate transaminase (AST) were measured using an enzymatically auto-analyzer (AU640, Olympus, Kobe, Japan) [22]. Blood samples were collected from each subject after a mandatory overnight fast lasting for 12 hours.

### 2.3 Substance Use, Physical Activity, and Physical Fitness

At the baseline health examination (2014), participants were requested to self-report their substance use status, including alcohol intake, betel nut chewing, and cigarette smoking in the past 6 months. This information was further classified into former or never against current active categories. In addition, participants also self-reported their moderate-intensity PA assessed by leisure time running, categorized as <150, 150-299, and ≥300 minutes per week in the past 6 months. The physical fitness of each participant was assessed through a 3000-m run test, which was performed outdoors at 16:00 P.M., contingent on favorable weather conditions in line with military testing regulations [23]. The entire procedure was video-recorded throughout the study.

### 2.4 Definition of MetS

Based on the recommendation by the International Diabetes Federation for the Chinese adult population [1], MetS was defined as the presence of three or more clinical features, which include: (1) waist circumference ≥90 cm in men and ≥ 80 cm in women; (2) systolic BP ≥130 mmHg, and/or diastolic BP ≥85 mmHg, or the use of antihypertensive medications; (3) serum triglycerides ≥150 mg/dL, or the use of lipid-lowering medications; (4) fasting glucose ≥100 mg/dL, or the use of antidiabetic medications; (5) and HDL-C <40 mg/dL in men and <50 mg/dL in women.

### 2.5 Definition of Hepatitis

As part of the annual health examination, participants with elevated ALT levels (≥27 U/L in men and ≥15 U/L in women) [19] of unknown cause undergo work-up, including serologic testing for hepatitis B surface antigen, anti-hepatitis C virus IgG, anti-hepatitis A virus IgM, and alpha-fetoprotein. Liver ultrasounds were also performed by certificated sonographers, and diffuse hepatic steatosis was defined by the presence of at least two of three findings: (1) significant liver-kidney contrast, (2) vascular blurring, and (3) deep attenuation of ultrasound signal according to the 2007 Asian Pacific Association for the Study of the Liver (APASL) guideline [24]. Since alcohol intake is strictly forbidden in military bases, military personnel are permitted to engage with alcoholic beverages solely during vacations. NAFLD was defined by ultrasound findings along with mildly elevated serum ALT levels ranging from 27 to 53.9 U/L in men and 15 to 29.9 U/L in women (1-fold to 2-fold of the suggested sex-specific upper limits). For NASH, the severe form of NAFLD, the diagnosis was made based on the ultrasound findings in conjunction with moderately elevated ALT levels ≥54 U/L in men and ≥40 U/L in women (≥2 x the suggested sex-specific upper limits). CHB was diagnosed by the presence of the hepatitis B surface antigen with a prior history [18].

### 2.6 Statistical Analysis

Four groups were classified as the unaffected individuals, NAFLD, NASH, and CHB from the cohort. The baseline characteristics of each group were revealed using mean ± standard deviation (SD) for continuous variables and compared by analysis of variance (ANOVA) and numbers (percentage) for categorical variables and compared by chi-squared test. Follow-up for each subject started at the baseline in 2014 and continued till the first incidence of MetS, loss to follow-up, or the end of the follow-up on December 31^st^, 2020. The survival analysis for incident MetS used Kaplan-Meier Curve analysis, and group differences were assessed using a log-rank test. Multivariable Cox regression analysis was used to examine the associations of NAFLD, NASH, and CHB with incident MetS. The stepwise analysis included a crude model and then simultaneous adjustments for baseline age, sex, service specialty, substance use, ALT, and BMI, which was treated as a categorical variable (< 25.0 kg/m^2^, 25.0 - 29.9 kg/m^2^ and ≥30 kg/m^2^) (Model 1). PA levels and physical fitness were further adjusted in Model 2. The associations of NAFLD, NASH, and CHB with an incidence of an individual feature of MetS were also explored utilizing multivariable Cox regression analysis with simultaneous adjustments for the covariates of Model 2.

We conducted stratified analyses to examine the associations between NAFLD, NASH, and CHB and the occurrence of MetS by taking BMI status into account (with *vs.* without overweight/obesity, defined as BMI ≥25.0 kg/m^2^). Formal tests for multiplicative interactions were conducted. A two-sided *P* value of less than 0.05 was considered as statistical significance. All statistical analyses were conducted using SPSS v26.0 software for Windows (IBM Corp., Armonk, NY, USA).

## 3 RESULTS

### 3.1 Participants Selected for Analysis

In the initial cohort of 4,080 individuals, we excluded participants with baseline MetS (n = 457), individuals aged 40 years or older (n =58), and those lost to follow-up due to relocation from the military bases in Eastern Taiwan (n =675). In addition, those who had a significant increase in ALT were further excluded (n =276). This resulted in a final sample of 2,614 subjects for analysis. Of those with mildly elevated ALT and free of CHB (N =645), liver sonographic findings for fatty infiltration were found in 426 participants (68.2%) who were classified to have NALFD, and the other 199 participants without sonographic evidence were grouped to the unaffected. Of those with moderately elevated ALT and free of CHB (N =104), sonographic fatty infiltrations of the liver were found in 78 participants (75.0%) who were classified to have NASH, and the other 26 ones without sonographic evidence were grouped to the unaffected. Of those with CHB (N =78), 36 participants were found with liver sonographic evidence for fatty infiltration (46.2%). The unaffected group included 1,807 participants with non-elevated ALT levels and 225 participants with elevated ALT while without sonographic evidence for fatty infiltration of the liver. Notably, in this cohort, no cases of viral hepatitis A, C, and hepatic malignancies were found.

### 3.2 Baseline Clinical Characteristics

Table **1** presents the baseline characteristics of the cohort. There were differences among the four groups in terms of age, sex, betel-nut chewing status, PA levels, physical fitness, BMI, waist circumference, SBP, DBP, and serum levels of total cholesterol, LDL-C, HDL-C, triglycerides, fasting glucose, uric acid, ALT, and AST. In general, the unaffected individuals were found to have fewer abnormal metabolic profiles than the NASH, NAFLD, and CHB groups.

### 3.3 Associations of NASH, NAFLD, and CHB with Incident MetS

**Fig. (1):**
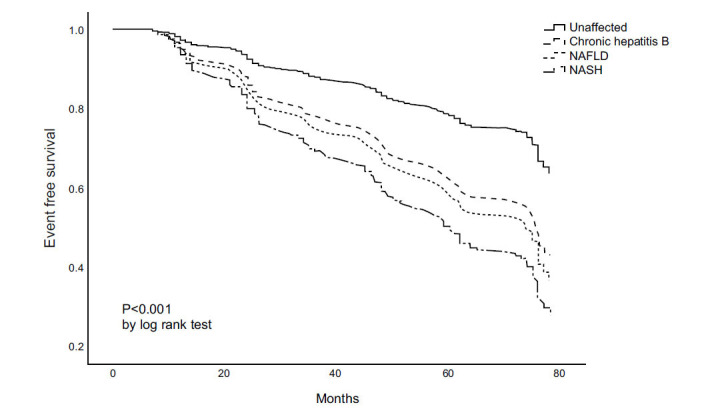
In the Kaplan-Meier curve analysis, a higher incidence of new-onset MetS in participants with NASH, NAFLD, and CHB as compared to unaffected participants was observed (from the highest to the lowest, *p* <0.01).

Over a median follow-up period of 5.8 years, there were 582 (22.3%) new-onset MetS events. (Fig. **1**) presents the results of the Kaplan-Meier curve analysis, with a higher incidence of new-onset MetS in participants with NASH, NAFLD, and CHB as compared to unaffected participants (from the highest to the lowest, *p* <0.001 by log-rank test).

Table **2** presents the results of multivariable Cox regression analysis for incident MetS while accounting for NASH, NAFLD, and CHB. Participants with NASH, NAFLD, and CHB exhibited an increased risk of MetS compared to those unaffected in the crude model. Upon adjusting for the baseline potential covariates, *e.g.,* PA levels and physical fitness in multivariable Model 1 and Model 2, the associations with NASH and NAFLD remained significant (hazard ratios (HRs) and 95% confidence intervals in Model 1: 1.76 (1.23-2.53) and 1.49 (1.22-1.81), respectively, and in Model 2: 1.66 (1.16-2.39) and 1.47 (1.21-1.79), respectively). On the contrary, CHB was no longer associated with new-onset MetS in Model 1 and Model 2 (HRs: 0.97 (0.61 - 1.54) and 0.97 (0.61-1.55), respectively).

### 3.4 Associations of NASH, NAFLD, and CHB with each MetS Feature

Table **3** presents the results of multivariable Cox regression analysis, exploring the associations of NASH, NAFLD, and CHB with incidences of the five MetS features in those free of the corresponding MetS feature at baseline. Both NASH and NAFLD were associated with a higher incidence of incident central obesity (HRs: 2.47 (1.54-3.96) and 1.47 (1.14-1.89), respectively), increased BP (HRs: 1.90 (1.28-2.84) and 1.60 (1.28-2.00), respectively), hypertriglycemia (HRs: 1.71 (1.10-2.66) and 1.38 (1.09-1.75), respectively), low HDL-C (HRs: 1.78 (1.19-2.66) and 1.54 (1.23-1.92), respectively), and prediabetes or diabetes (HRs: 1.57 (1.04-2.37) and 1.45 (1.17-1.81), respectively). However, no association between CHB and the incidence of each MetS feature was observed (HRs from 0.78 to 1.05).

### 3.5 Associations of NASH, NAFLD, and CHB with Incident MetS by BMI Status

Table **4** presents the results of multivariable Cox regression analysis stratified by BMI status. Regardless of BMI ≥25 kg/m^2^ or <25 kg/m^2^ at baseline, participants with NASH and NAFLD had a greater risk of new-onset MetS (HRs for NASH: 1.57 (1.04-2.36) and 2.53 (1.17-5.45), respectively, *p*-value for interaction =0.29; and HRs for NAFLD: 1.34 (1.07-1.70) and 2.19 (1.52-3.16), respectively, *p*-value for interaction =0.02). Those with CHB did not have a greater risk of new-onset MetS as compared to the unaffected individuals, regardless of BMI ≥25 kg/m^2^ or <25 kg/m^2^ at baseline (HRs: 1.03 (0.61–1.74) and 0.91 (0.36–2.29), respectively, *p*-value for interaction: 0.67). Notably, there is a tendency that the associations of NASH and NAFLD with incident MetS were relatively lower in participants with BMI ≥25 kg/m^2^ than in those with BMI <25 kg/m^2^. On the contrary, the association of CHB was relatively greater in those with BMI ≥25 kg/m^2^ than in those with BMI <25 kg/m^2^, despite statistically nonsignificance.

### 3.6 Effects of the Presence of NASH and NAFLD in those with CHB

Table **S1** reveals the effects of the presence of NASH and NAFLD on incident MetS in those with CHB. In those simultaneously with NASH or NAFLD, the incidence of MetS increased (HR: 1.15 (0.67-1.99)), while in those without NASH or NAFLD, the incidence of MetS decreased (HR: 1.15 (0.67-1.99)). Since the sample size of those with CHB was small, the statistical power was not sufficient to detect the difference between the two subgroups.

## 4 DISCUSSION

Our principal findings were that among young adults, CHB was not associated with incident MetS and the related features with adjustments for the potential covariates, including the crucial confounders, *i.e.,* PA levels and physical fitness. In addition, NAFLD and NASH were associated with a greater risk of incident MetS, which was in line with the findings in prior studies. Notably, the strength of the association for incident MetS was greater in participants with NASH, the severe form of NAFLD, than those with non-severe NAFLD. Furthermore, the strength of the association for incident MetS with NAFLD and NASH seems to be greater in those with normal weight than those with overweight or obesity, whereas for CHB, the condition seems to be reversed.

Growing evidence supports an association between MetS and NAFLD [5, 18]. Pathogenesis seems to have common pathophysiological mechanisms that involve cellular metabolism and insulin resistance as key factors. In NAFLD or NASH, dysfunctional adipose tissue impacts MetS progression by releasing cytokines that contribute to systemic insulin resistance [25]. The inflammatory response operates through two pathways: the nuclear factor Kappa beta (NFκβ) and c-Jun NH2 terminal kinase (JNK) signaling pathways [26, 27]. In addition, the presence of hypoadiponectinemia and oxidative stress accompanying NAFLD also leads to metabolic derangement [28, 29]. Many cross-sectional studies have confirmed the association of ALT levels and NAFLD with prevalent MetS [11-13]. Our findings were in line with the prior studies and indicated a dose-response association between higher serum ALT levels reflecting more severe NAFLD and the increased risk of new-onset MetS among young adults.

The association between CHB and MetS remains not fully understood. Current evidence for this association was inconsistent. In an early cross-sectional study involving 53,528 subjects in Taiwan, an inverse association between CHB and prevalent MetS was found. Similarly, in a large U.S. population database, the Third National Health and Nutrition Examination Survey (NHANES III), there was a lower prevalent MetS among individuals with CHB as compared to the general population, with an adjusted odds ratio (OR) of 0.32 [30]. In addition, a meta-analysis also found a lower prevalence (OR: 0.80) of MetS in CHB subjects compared to healthy controls. Based on evidence from these cross-sectional studies, the possibility of MetS might be lower in those with CHB, particularly among males, those aged under 45 years, and lean individuals [6]. However, a 20-year follow-up cohort study unveiled a higher risk of incident MetS in patients with CHB compared to the unaffected individuals (HR: 2.27) with adjustments for potential covariates, including baseline PA [31, 32]. In addition, a cross-sectional study showed a greater possibility of MetS with CHB in women (adjusted OR: 1.23). Our study was the first cohort study to include information on baseline PA and physical fitness, which were utilized for adjustments in the model to verify a null temporal association between CHB and incident MetS in young adults. Mechanisms for the neutral or lower MetS risk with CHB have been proposed by an increase in adiponectin, which has anti-inflammation, anti-oxidation, anti-fibrosis, and immunomodulation effects [33]. In addition, CHB infection has been associated with a lower risk of elevated BP and dyslipidemia, although a higher risk of insulin resistance and type 2 diabetes with CHB was observed in some specific subgroups [34]. The effect of overweight or obesity on CHB for the increased risk of cardiometabolic comorbidities was also found in this study, despite statistically non-significance [35].

This study had some limitations. Firstly, since the diagnosis of NASH required pathologic findings, which were not available in this study, we used both higher serum ALT and the presence of sonographic findings as a surrogate of NASH [36]. Second, the predominance of male participants (90.8%) in our study cohort may limit the generalization of our findings to the broader population. Third, since sonography was only performed in participants with elevated serum ALT or CHB, many participants with NAFLD might have a normal ALT and be classified as the unaffected group, possibly underestimating the risk of incident MetS with CHB. Fourth, we did not use the specific criteria for the Taiwanese to define the normal weight (BMI: 18.5-23.9 kg/m^2^) and overweight or obesity (BMI ≥24.0 kg/m^2^) since there were too few events during the follow-up period to evaluate the risk. Fifth, the participants' dietary and living habits, which may affect fatty liver diseases and body mass, were not reported, possibly resulting in a bias. Despite the limitations, this study exhibits some strengths. Firstly, the homogeneity of military personnel in terms of living environments and training helps to mitigate the impact of unaccounted confounders. Second, both PA and physical fitness were taken in the multivariable models that have been widely acknowledged with a substantial link to MetS while neglected in most of the prior studies. Third, in the context of the association between CHB and MetS, prior studies have primarily relied on cross-sectional analyses. Our study was the first cohort study to investigate the temporal association between CHB and incident MetS before midlife among young adults.

## CONCLUSION

In summary, this study demonstrated that in young adults, CHB was not a risk factor of incident MetS with adjustments for potential covariates, including PA and aerobic fitness at baseline, whereas NAFLD and NASH were independent risk factors of new-onset MetS in young adults, which were in line with prior study findings.

## Figures and Tables

**Table 1:** Baseline clinical characteristics (n = 2,614).

-	**NAFLD (n = 426)**	**NASH (n = 78)**	**CHB (n = 78)**	**Unaffected (n = 2,032)**	***p* Value**
Age, years	29.75 ± 5.55	29.53 ± 5.08	32.53 ± 3.62	27.72 ± 5.70	< 0.001
Male sex, %	381 (89.1)	76 (97.4)	74 (94.9)	1842 (90.6)	0.08
Specialty, %	-	-	-	-	-
Army	198 (46.5)	35 (44.9)	33 (42.3)	1073 (52.8)	0.12
Navy	106 (24.9)	21 (26.9)	20 (25.6)	431 (21.2)	-
Air force	122 (28.6)	22 (28.2)	25 (32.1)	528 (26.0)	-
Active substance used, %	-	-	-	-	-
Alcohol drinking	191 (44.8)	33 (42.3)	36 (46.2)	793 (39.0)	0.10
Betel-nut chewing	61 (14.5)	11 (14.1)	5 (6.4)	174 (8.7)	0.001
Cigarette smoking	155 (36.7)	29 (37.2)	21 (26.9)	689 (34.3)	0.36
PA levels, %	-	-	-	-	-
<150 min/wk	79 (18.5)	9 (11.5)	15 (19.2)	422 (20.8)	0.04
150 – 299 min/wk	179 (42.0)	41 (52.6)	25 (32.1)	760 (37.4)	-
≥300 min/wk	168 (39.4)	28 (35.9)	38 (48.7)	850 (41.8)	-
3000-m run test, seconds	887.61 ± 85.95	887.85 ± 69.45	874.91 ± 74.87	863.34 ± 79.80	< 0.001
BMI, kg/m^2^	25.67 ± 2.82	26.61 ± 2.52	24.95 ± 2.54	23.71 ± 2.86	< 0.001
18.5 – 25.0 kg/m^2^	181 (42.5)	25 (32.1)	39 (50.0)	1394 (66.6)	< 0.001
25.0 – 29.9 kg/m^2^	212 (49.8)	47 (60.3)	36 (46.2)	602 (29.6)	-
≥30.0 kg/m^2^	33 (7.7)	6 (7.7)	3 (3.8)	36 (1.8)	-
Waist circumferences, cm	85.21 ± 7.83	87.89 ± 6.01	83.48 ± 6.60	79.99 ± 7.67	< 0.001
SBP, mmHg	117.63 ± 12.89	120.35 ± 11.28	117.82 ± 11.98	115.40 ± 12.78	< 0.001
DBP, mmHg	70.63 ± 9.73	71.19 ± 9.95	72.88 ± 10.21	68.68 ± 9.46	< 0.001
Blood test	-	-	-	-	-
Total cholesterol, mg/dL	184.08 ± 32.92	192.97 ± 36.72	181.58 ± 41.25	167.39 ± 30.09	< 0.001
LDL-C, mg/dL	114.24 ± 29.40	122.28 ± 30.33	112.22 ± 33.35	99.66 ± 27.04	< 0.001
HDL-C, mg/dL	48.45 ± 9.81	48.01 ± 9.38	47.40 ± 9.75	50.63 ± 10.05	< 0.001
Triglycerides, mg/dL	121.59 ± 79.29	130.68 ± 95.34	104.50 ± 77.44	88.74 ± 47.06	< 0.001
FG, mg/dL	93.07 ± 9.13	92.22 ± 10.91	92.19 ± 7.71	91.68 ± 9.33	0.04
UA, mg/dL	6.77 ± 1.44	7.46 ± 1.43	6.45 ± 1.21	6.38 ± 1.29	< 0.001
ALT, U/L	33.25 ± 8.44	79.50 ± 29.60	33.18 ± 32.41	14.82 ± 5.16	< 0.001
AST, U/L	25.35 ± 8.08	41.14 ± 16.46	24.50 ± 15.73	17.55 ± 4.60	< 0.001

**Note: ** Continuous variables were shown as mean (standard deviation) and categorical variables were shown as n (%). **Abbreviations:** PA, physical activity; BMI, body mass index; SBP, systolic blood pressure; DBP, diastolic blood pressure; LDL-C, low-density lipoprotein cholesterol; HDL-C, high-density lipoprotein cholesterol; FG, fasting glucose; UA, uric acid; ALT, alanine transaminase; AST; aspartate transaminase.

**Table 2:** Associations of nonalcoholic fatty liver disease, nonalcoholic steatohepatitis and chronic hepatitis B with new-onset MetS.

-	-	-	**Crude Model**		**Model 1**		**Model 2**	
	**N**	**MetS Events**	**HR (95% CI)**	***p* Value**	**HR (95% CI)**	***p* Value**	**HR (95% CI)**	***p* Value**
NASH	78	33	2.87 (2.01 - 4.10)	< 0.001	1.76 (1.23 - 2.53)	0.002	1.66 (1.16 - 2.39)	0.006
NAFLD	426	154	2.22 (1.84 - 2.69)	< 0.001	1.49 (1.22 - 1.81)	< 0.001	1.47 (1.21 - 1.79)	< 0.001
CHB	78	26	1.97 (1.33 - 2.94)	0.001	0.97 (0.61 - 1.54)	0.89	0.97 (0.61 - 1.55)	0.91
Unaffected (reference)	2032	369	1.00	-	1.00	-	1.00	-

**Note: ** Data are presented as hazard ratio (HR) and 95% confidence interval (CI) using multivariable Cox regression analysis with adjustments for age, sex, specialty, substance use, alanine transaminase and BMI levels in Model 1. In Model 2, besides adjusting for the confounding factors in Model 2, there is an additional adjustment for both physical activity levels and physical fitness. **Abbreviations:** NASH, nonalcoholic steatohepatitis; NAFLD, nonalcoholic fatty liver disease; CHB, chronic hepatitis B; MetS, metabolic syndrome.

**Table 3:** Associations of Nonalcoholic fatty liver disease, nonalcoholic steatohepatitis and chronic hepatitis b with new-onset central obesity, hypertension, dyslipidemia and prediabetes/diabetes.

-	-	-	**NASH**		**NAFLD**		**CHB**	
	**n**	**Events**	**HR (95% CI)**	***p* Value**	**HR (95% CI)**	***p* Value**	**HR (95% CI)**	***p* Value**
Central obesity	2,388	542	2.47 (1.54 - 3.96)	< 0.001	1.47 (1.14 - 1.89)	0.003	1.05 (0.62 - 1.79)	0.85
Hypertension	2,434	476	1.90 (1.28 - 2.84)	0.002	1.60 (1.28 - 2.00)	< 0.001	0.78 (0.44 - 1.39)	0.39
Hypertriglyceridemia	2,553	551	1.71 (1.10 - 2.66)	0.01	1.38 (1.09 - 1.75)	0.007	0.91 (0.53 - 1.55)	0.72
Low HDL-C	2,457	377	1.78 (1.19 - 2.66)	0.005	1.54 (1.23 - 1.92)	< 0.001	0.87 (0.51 - 1.48)	0.60
Prediabetes/diabetes	2,530	503	1.57 (1.04 - 2.37)	0.03	1.45 (1.17 - 1.81)	0.001	1.01 (0.61 - 1.68)	0.96

**Note: ** Data are presented as hazard ratio (HR) and 95% confidence interval (CI) using multivariable Cox regression analysis with adjustments for age, sex, specialty, substance use, body mass index levels, alanine transaminase, physical activity levels and physical fitness. **Abbreviations:** NASH, nonalcoholic steatohepatitis; NAFLD, nonalcoholic fatty liver disease; CHB, chronic hepatitis B; HDL-C, high density lipoprotein cholesterol. Central obesity was defined as waist circumference ≥90 cm in men and ≥80 cm in women. Low HDL-C was defined as HDL-C <40 cm in men and <50 cm in women.

**Table 4:** Associations of nonalcoholic fatty liver disease, nonalcoholic steatohepatitis and chronic hepatitis B with new-onset MetS stratified by body mass index levels.

-	**BMI ≥25.0 kg/m^2^**				**BMI <25.0 kg/m^2^**				***P* Value for Interaction**
	**N**	**MetS Events**	**HR (95% CI)**	***p* Value**	**N**	**MetS Events**	**HR (95% CI)**	***p* Value**	
NASH	53	26	1.57 (1.04 – 2.36)	0.03	25	7	2.53 (1.17 – 5.45)	0.01	0.29
NAFLD	245	115	1.34 (1.07 – 1.70)	0.01	181	39	2.19 (1.52 – 3.16)	<0.001	0.02
CHB	39	21	1.03 (0.61 – 1.74)	0.90	39	5	0.91 (0.36 – 2.29)	0.84	0.67
Unaffected (reference)	638	236	1.00	-	1394	133	1.00	-	-

**Note: ** Data are presented as hazard ratio (HR) and 95% confidence interval (CI) using multivariable Cox regression analysis with adjustments for age, sex, specialty, substance use, body mass index levels, alanine transaminase, physical activity levels and physical fitness. **Abbreviations:** BMI, body mass index; NASH, nonalcoholic steatohepatitis; NAFLD, nonalcoholic fatty liver disease; CHB, chronic hepatitis B; MetS, metabolic syndrome.

## Data Availability

This study used data from the Cardiorespiratory Fitness and Hospitalization Events in the Armed Forces (CHIEF). The data that support the findings of this study are available from the corresponding author upon reasonable request.

## LIST OF ABBREVIATIONS

CHIEF: Cardiorespiratory Fitness and Health in Eastern Armed Forces
LDL-C: Low-Density Lipoprotein Cholesterol
MetS: Metabolic Syndrome
NAFLD: Nonalcoholic Fatty Liver Disease
NFκβ: Nuclear Factor Kappa Beta
NHANES III: Third National Health and Nutrition Examination Survey
PA: Physical Activity
SD: Standard Deviation

## STANDARDS OF REPORTING

STROBE guidelines were followed.
