# Supplementary material for: Associations of Chronic Hepatitis B and Nonalcoholic Fatty Liver Diseases with New-Onset Metabolic Syndrome in Military Personnel before Midlife: A Cohort Study
Source: Endocr Metab Immune Disord Drug Targets. 2025 Jan 9;25(13):1073–81. doi: 10.2174/0118715303323078241022050619 (PMC12715392; doi:10.2174/0118715303323078241022050619)
Supplement: Supplementary file 1 [file EMIDDT-25-13-1073_SD1.pdf]

Supplementary Material

Associations of Chronic Hepatitis B and Nonalcoholic Fatty Liver Diseases with New-Onset Metabolic Syndrome in Military Personnel before Midlife: A Cohort Study

Kun-Zhe Tsai[1,2,3], Pang-Yen Liu[4], Yen-Chen Lin[5], Chen-Ming Huang[6], Hui-Shang Wang[7] and Gen-Min Lin[1,4,*]

[1]Department of Medicine, Hualien Armed Forces General Hospital, Hualien City, Taiwan; [2]Department of Stomatology of Periodontology, Mackay Memorial Hospital, Taipei, Taiwan; [3]Department of Periodontology, School of Dentistry, National Defense Medical Center and Tri-Service General Hospital, Taipei, Taiwan; [4]Department of Medicine, Tri-Service General Hospital, National Defense Medical Center, Taipei, Taiwan; [5]Department of Medicine, Linkou Chang Gung Memorial Hospital, Taoyuan, Taiwan; [6]Department of Cardiovascular Surgery, Mennonite Christian Hospital, Hualien City, Taiwan; [7]Division of Cardiology, Department of Internal Medicine, Hualien Tzu Chi General Hospital, Hualien City, Taiwan

Table S1. The Effect of NAFLB and NASH on the Incidence of Metabolic Syndrome in Participants With CHB

	**With NAFLB or NASH simultaneously**					**Without NAFLB or NASH**				***P* value for interaction**
	**N**	**MetS events**	**HR (95% CI)**	***p* value**		**N**	**MetS events**	**HR (95% CI)**	***p* value**	
CHB	28	16	1.15 (0.67 – 1.99)	0.60		50	10	0.70 (0.37 – 1.35)	0.28	0.10
Unaffected (reference)	568	240	1.00			1968	316	1.00		

Note: data are presented as hazard ratio (HR) and 95% confidence interval (CI) using multivariable Cox regression analysis with adjustments for age, sex, specialty, substance use, body mass index levels, alanine transaminase, physical activity levels and physical fitness. **Abbreviations:** NASH, nonalcoholic steatohepatitis; NAFLD, nonalcoholic fatty liver disease; CHB, chronic hepatitis B; MetS, metabolic syndrome
